# *Azorhizobium caulinodans* Transmembrane Chemoreceptor TlpA1 Involved in Host Colonization and Nodulation on Roots and Stems

**DOI:** 10.3389/fmicb.2017.01327

**Published:** 2017-07-13

**Authors:** Wei Liu, Jinbao Yang, Yu Sun, Xiaolin Liu, Yan Li, Zhenpeng Zhang, Zhihong Xie

**Affiliations:** ^1^Key Laboratory of Coastal Biology and Bioresource Utilization, Yantai Institute of Coastal Zone Research, Chinese Academy of Sciences Yantai, China; ^2^College of Life Sciences, Shanxi Agricultural University Taigu, China; ^3^School of Resource and Environment, University of Chinese Academy of Sciences Beijing, China

**Keywords:** chemotaxis, *Azorhizobium caulinodans*, chemoreceptor, symbiosis, nodulation

## Abstract

*Azorhizobium caulinodans* ORS571 is a motile soil bacterium that interacts symbiotically with legume host *Sesbania rostrata*, forming nitrogen-fixing root and stem nodules. Bacterial chemotaxis plays an important role in establishing this symbiotic relationship. To determine the contribution of chemotaxis to symbiosis in *A. caulinodans* ORS571-*S. rostrata*, we characterized the function of TlpA1 (transducer-like protein in *A. caulinodans*), a chemoreceptor predicted by SMART (Simple Modular Architecture Research Tool), containing two N-terminal transmembrane regions. The *tlpA1* gene is located immediately upstream of the unique *che* gene cluster and is transcriptionally co-oriented. We found that a Δ*tlpA1* mutant is severely impaired for chemotaxis to various organic acids, glycerol and proline. Furthermore, biofilm forming ability of the strain carrying the mutation is reduced under certain growth conditions. Interestingly, competitive colonization ability on *S. rostrata* root surfaces is impaired in the Δ*tlpA1* mutant, suggesting that chemotaxis of the *A. caulinodans* ORS571 contributes to root colonization. We also found that TlpA1 promotes competitive nodulation not only on roots but also on stems of *S. rostrata*. Taken together, our data strongly suggest that TlpA1 is a transmembrane chemoreceptor involved in *A. caulinodans*-*S. rostrata* symbiosis.

## Introduction

Bacterial motility and chemotaxis are thought to play an important role in adaptation to soil and rhizosphere. Chemotaxis is an important factor in establishing the initial chemical and physical contact between the bacteria and host roots (Caetano-Anollés et al., 1988b; Miller et al., 2007; Scharf et al., 2016). Chemotaxis towards root exudates has been suggested to be the first step in bacterial colonization of plant roots (Vande Broek and Vanderleyden, 1995; Gupta, 2003; Greer-Phillips et al., 2004). Bacterial biofilm formation also contributes to effective colonization of plant roots (Timmusk et al., 2005; Li et al., 2013). In the rhizobia-legume association, chemotaxis towards chemoattractants released from roots can promote infection and nodulation by guiding rhizobia to proper site for nodulation (Ames and Bergman, 1981; Bauer and Caetano-Anollés, 1990; Greer-Phillips et al., 2004). Chemoreceptors allow bacteria to detect attractants and repellents, thereby directing bacteria to find favorable conditions (Broek and Vanderleyden, 1995). Previous research has suggested that various species of rhizobia show chemotaxis toward plant root or seed exudates (Aguilar et al., 1988; Caetano-Anollés et al., 1988a; Barbour et al., 1991; Webb et al., 2014, 2017) and that certain mutants impaired for chemotaxis are also less competitive than wild-type in root colonization and nodulation (Dharmatilake and Bauer, 1992; Yost et al., 1998; Miller et al., 2007; Jiang et al., 2016a).

Motile bacteria such as *Escherichia coli* regulate chemotactic behavior based on environmental signals by a two-component signal transduction network. The molecular mechanism of chemotaxis has been best studied in the model organism, *E. coli*, which encodes a single chemotaxis system comprised of a histidine kinase CheA and its response regulator CheY (Falke et al., 1997). CheA is coupled to the transmembrane chemoreceptor by CheW scaffold protein. Transmembrane chemoreceptors provide input for initiation of the chemotaxis signal transduction cascade (Wuichet et al., 2007) and regulate cell motility by modulating histidine kinase activity of CheA. Phosphorylated CheA transfers its phosphoryl group to CheY, forming phospho-CheY, which influences the rotational direction of the flagella (Hazelbauer et al., 2008). However, chemosensory signaling systems in many other bacteria, including plant-associated bacteria, are more complex. Many motile bacteria possess two or more chemotaxis systems and several chemoreceptors (Mukherjee et al., 2016). Chemoreceptors typically contain two distinct structural and functional domains: a variable N-terminal periplasmic ligand-binding domain (LBD), which is responsible for sensing environmental stimuli, and a conserved C-terminal domain that transmits signals to the downstream chemotactic machinery. The C-terminal domain of membrane-bound chemoreceptor can be further differentiated into a HAMP (Histidine kinases, Adenylyl cyclases, Methyl-accepting proteins and Phosphatases) domain and a MA (methyl-accepting chemotaxis-like) domain. The HAMP domain transmits transmembrane ligand binding signals to the MA domain (Collins et al., 2014). The MA domain interacts with CheW and CheA (Zhulin, 2001; Collins et al., 2014).

*Azorhizobium caulinodans* ORS571 is a motile alpha-proteobacterium, capable of fixing nitrogen. It can also form a nitrogen-fixing symbiotic relationship with the host plant *Sesbania rostrata* (Trinick, 1979; Dreyfus et al., 1983, 1988; Tsien et al., 1983), which is a small semi-aquatic leguminous tree (Cook et al., 2005; Capoen et al., 2010). This symbiotic relationship leads to the formation of nitrogen fixing nodules in the *S. rostrata* (Capoen et al., 2010). While nodules normally occur on roots of leguminous plants, stem nodules can also be formed at the site of adventitious root primordia following crack-entry invasion by *A. caulinodans* (Tsien et al., 1983). Chemoreceptors of nitrogen-fixing rhizobia play a crucial role in establishing a symbiotic relationship with the host plant (Yost et al., 1998).

We used the MiST2 (Microbial Signal Transduction) and SMART (Simple Modular Architecture Research Tool) databases to identify 43 chemoreceptors in *A. caulinodans* ORS571 (Jiang et al., 2016b) based on its sequenced genome (Lee et al., 2008). Of these, 30 were localized to the cytoplasmic membrane fraction as they were predicted to contain two transmembrane spanning regions. Four of the chemoreceptors contained only one transmembrane domain, while nine internal chemotaxis proteins lacked a transmembrane domain. Jiang et al. (2016a) have identified a PAS-containing soluble internal chemotaxis protein IcpB in *A. caulinodans* that is important for nodulation and nitrogen fixation upon symbiosis with *S. rostrata* (Jiang et al., 2016a). In this work, we characterize a transmembrane chemoreceptor TlpA1 in *A. caulinodans* and describe its role in chemotaxis and competitive nodule formation on roots and stems of host plants.

## Materials and Methods

### Strains and Plasmids

All bacterial strains and plasmids used in this study are listed Supplementary Table S1. Wild-type *A. caulinodans* ORS571 and its mutant derivatives were grown in L3 minimal medium (Jiang et al., 2016a), TY medium or on agar plates at 37°C containing 25 μg ml^-1^ nalidixic acid and 100 μg ml^-1^ ampicillin. *E. coli* was grown in LB broth or on LB agar plates at 37°C with the following concentrations of antibiotics as required: 50 μg ml^-1^ kanamycin, 100 μg ml^-1^ ampicillin, 50 μg ml^-1^ gentamycin, 10 μg ml^-1^ tetracycline.

### Generation of Mutant and Complemented Strains

A *tlpA1*-deficient strain was constructed as follows: A 693-bp fragment spanning a region immediately upstream of *tlpA1* and the 5′ end of the gene (Supplementary Figure S1A) was amplified by PCR from the genomic DNA of *A. caulinodans* ORS571 using primer pair TlpA1UF and TlpA1UR (Supplementary Table S2). A 653-bp fragment encompassing 501-bp at 3′ end of *tlpA1*, the stop codon and a 152-bp region immediately downstream of the gene (Supplementary Figure S1A) was amplified from the genomic DNA of the wild-type strain using the primer pair TlpA1DF and TlpA1DR (Supplementary Table S2). The 693-bp fragment was digested with KpnI and NdeI and ligated into KpnI and NdeI sites of the vector pCM351 (Marx and Lidstrom, 2002). One of the resulting recombinant plasmid was designated as pCM351::UF. The 653-bp fragment was digested with ApaI-SacI enzymes and cloned into ApaI-SacI sites of the recombinant plasmid pCM351::UF. The ligation mixtures were transformed into *E. coli* DH5α to recover individual clones. One of the resulting recombinant plasmids, designated as pCM351::UF::DF, was verified by sequencing. The recombinant plasmid pCM351::UF::DF was transferred into wild-type *A. caulinodans* ORS571 by tri-parental conjugation with a helper plasmid pRK2013 (Figurski and Helinski, 1979). Colonies were screened for loss of the recombinant plasmid and for double homologous recombination. Potential mutants were obtained from on TY plates by selecting for gentamicin-resistance (from pCM351 vector) and tetracycline-sensitivity (Marx and Lidstrom, 2002). The gentamicin gene was then deleted by introduction of plasmid pCM157 (Marx and Lidstrom, 2002). Mutations in the strains were verified by PCR with primer pair TlpA1F and TlpA1R. A confirmed mutation was named Δ*tlpA1*.

For complementation, the *tlpA1* ORF with 737-bp upstream non-coding sequence was amplified by PCR using primer pair TlpA1comF and TlpA1comR (Supplementary Table S2). The amplified 2250-bp fragment was inserted into HindIII and BamHI sites of the broad-host-range cloning vector pBBR1MCS-2 (Kovach et al., 1995). The ligation mixture was transformed into *E. coli* DH5α. DNA sequences from individual clones were sequenced and one verified clone was designated as pBBR1MCS-2-*tlpA1*-com. This recombinant plasmid was then introduced into the *tlpA1* mutant via tri-parental mating and the transformants were recovered by selection for kanamycin resistance. One of the resulting strains was designated as *tlpA1*-com.

To construct a *cheA* deletion mutant of *A. caulinodans* ORS571, a 696-bp upstream fragment (UF) was amplified by PCR using primer pair CheAUF and CheAUR (Supplementary Table S2), and a 675-bp downstream fragment (DF) was amplified by PCR using primer pair CheADF and CheADR (Supplementary Table S2). The upstream PCR product was digested with NsiI-NdeI and cloned into NsiI-NdeI sites of vector pCM351. The resulting plasmid was designated as pCM351::UF. The downstream PCR product was digested with AgeI-SacI and cloned into AgeI-SacI sites of pCM351::UF. The final plasmid pCM351::UF::DF was recovered from the ligation mixture by transforming into *E. coli* DH5α and verified by sequencing. The plasmid was transferred into *A. caulinodans* ORS571 by a tri-parental mating with the aid of a helper plasmid pRK2013. The gentamicin gene replaces the *cheA* gene by double homologous recombination in the potential mutant, which was then removed by introducing the vector pCM157 (Marx and Lidstrom, 2002). The *cheA-*deletion mutant was thus generated by deletion of an internal 2340-bp fragment (Supplementary Figure S1B) and was verified by PCR with primer pair CheAF and CheAR. The confirmed mutant was named Δ*cheA*.

### Growth Experiments

Strains were grown overnight in TY medium containing 25 μg ml^-1^ nalidixic acid and 100 μg ml^-1^ ampicillin. Cultures were diluted either into L3 medium containing 10 mM succinate and 10 mM NH_4_Cl or into TY medium to an initial OD_600_ of 0.02 and grown at 200 rpm and 37°C. All data were depicted as means and standard deviations from three individual repetitions.

### Soft Agar Chemotaxis Assay

For the soft agar assay, overnight cultures were washed and resuspended in chemotaxis buffer (Jiang et al., 2016a). The cultures were then adjusted to an optical density at 600 nm (OD_600_) of 1.0. To compare chemotaxis response to different carbon sources, 5 μl aliquots of cell suspensions of the wild-type, mutant and complemented strains were inoculated on L3 soft agar plates (0.3% agar) containing 10 mM various carbon sources (succinate, citrate, proline, tartrate, malate, and glycerol) with or without 10 mM NH_4_Cl. The inoculated plates were incubated for three days at 37°C.

### Competitive Quantitative Capillary Chemotaxis Assay

A quantitative capillary chemotaxis assay was carried out as described in Reyes-Darias et al. (2016) with a few modifications. The wild-type and Δ*tlpA1* strains were grown overnight in TY medium at 200 rpm and 37°C. The cells were then washed with chemotaxis buffer and the cell pellets were resuspended to a final density of OD_600_ = 0.05. 200 μl of bacterial suspension mixtures (containing 100 μl wild-type and 100 μl mutant) were added into each well of a 96-well plate. Open ends of the capillaries containing chemotaxis buffer or 10 mM succinate as carbon source were then inserted into the wells. After incubation for an hour, the portions of the capillaries that were in contact with bacteria were rinsed with sterile water. The sealed ends were broken and the contents were transferred into microfuge tubes containing 1 ml sterile water. From each of these tubes, five individual 20 μl aliquots were placed onto TY plates and incubated at 37°C for 48 h prior to cell counting. Cells on TY plates were tested by PCR using primer pair TlpA1F and TlpA1R.

### Real-time PCR

Total RNA was isolated from cultured free-living cells. cDNA was generated using GoScript^TM^ Reverse Transcription Kit (Promega) and diluted 10-fold (analysis of *16S rRNA* expression) and 500-fold (analysis of the *cheA* gene expression) for subsequent PCR amplification. Quantitative PCR was performed using GoTaq^®^ qPCR Master Mix kit (Promega) with gene-specific primer pairs (TlpA1QF and TlpA1QR for *tlpA1*, CheAQF and CheAQR for *cheA*, 16SQF and 16SQR for *16S rRNA*). Genomic DNA of ORS571 strain was used as template to construct a standard curve for determination of gene copy numbers. Transcript levels of the *cheA* gene were normalized against *16S rRNA* levels. Real-time PCR was carried out on a Bio-Rad CFX96^TM^ real-time PCR system. The program for amplification included an initial denaturation step at 95°C for 2 min, followed by 40 cycles of 95°C for 10 s and 60°C for 30 s. Specificity of the reaction was monitored by melting curve analysis.

### Biofilm Formation Assay

Single colonies were inoculated into L3 liquid medium and incubated at 37°C and 200 rpm. The cell culture (OD_600_ around 2.5) was washed once with L3 medium and resuspended in an equal volume of L3 medium. For biofilm formation assay, 1.5 ml L3 medium was added to each glass tube, into which 150 μl cell cultures was added and incubated at 37°C for 3–5 days. For Crystal Violet (CV) staining, cultures were removed carefully with a pipette, and the tubes were washed 3–5 times with ddH_2_O and 1.6 ml of 0.5% CV was added into each tube. After incubation for 20 min, CV was removed and the tubes were washed five times with ddH_2_O. Finally, 1.5 ml of 30% acetic acid was added to each tube to wash off the “CV ring”. The OD value of each tube was determined at 570 nm after CV staining.

### Root Adsorption Assay of *S. rostrata* Seedlings

*Sesbania rostrata* seeds were treated as described previously (Suzuki et al., 2007) with a few modifications. Seeds were immersed in concentrated sulfuric acid for 30 min to induce uniform germination, rinsed five times with sterile water, and soaked in sterile water for two days at 37°C in dark. Cells were grown in TY medium and harvested by centrifugation at mid-log phase (OD_600_ of 0.5–0.6). The cells were washed thrice and resuspended in sterile chemotaxis buffer (Greer-Phillips et al., 2004) to an OD_600_ of 0.5. The concentration of the cell suspensions (OD_600_ = 0.5) was further verified by serial dilution and plating onto TY plates. 50 ml cultures of each strain were used to soak (50 rpm, 28°C) two day-old seedlings of *S. rostrata* for 4 and 24 h. The seedlings were then washed four times with sterile water at 100 rpm for 1 min each in a rotary bath. Equal-sized roots from five seedlings were then dispersed in sterile water by using a homogenizer. Serial dilutions from this suspension were spread onto TY agar plates and incubated for two days at 37°C. The adsorption pattern of root surface was examined by analysis of the wild-type and Δ*tlpA1* colonies on TY plates. In competition adsorption experiments, surface-sterilized seedlings were co-inoculated with wild-type ORS571 and Δ*tlpA1* strains at approximately 1:1 and 1:10 ratios for 4 and 24 h. Adsorbed bacteria were detected by PCR using the primer pair TlpA1F and TlpA1R. A non-inoculated flask was used as a control for all experiments.

### Nodulation and Competitive Nodulation Assays

Nodulation and competitive nodulation assays of *S. rostrata* were performed as previously described in Yost et al. (1998) with a few modifications. For nodulation assays on roots, surface-sterilized seedlings were co-inoculated with wild-type ORS571 or Δ*tlpA1* strains at an OD_600_ of approximately 0.5. For nodulation assays on stem, *S. rostrata* seeds were germinated and the seedlings were transferred to vermiculite in moistened pots containing a low-N nutrient (Callow and Vincent, 1971). After 3–4 weeks of plant growth, *S. rostrata* stems were inoculated with either the wild-type or Δ*tlpA1* strains at an OD_600_ of approximately 0.5. For nodulation competition assays on roots and stems, surface-sterilized seedlings and stems were co-inoculated with the wild-type ORS571 and Δ*tlpA1* mixtures at approximately 1:1 and 1:10 ratios. All *S. rostrata* plants were grown in the greenhouse at 27°C. Nodules were harvested 3 weeks post inoculation and bacteria were re-isolated from root and stem nodules. Competitive abilities of the wild-type and Δ*tlpA1* strains were analyzed by PCR using the primer pair TlpA1F and TlpA1R. Non-inoculated plants served as controls.

### Acetylene Reduction Activities (ARAs) of Nodules

To measure symbiotic ARA, 50 root or stem nodules were weighed and immediately placed into individual 3 ml sealed tubes. 300 μl acetylene (C_2_H_2_) gas was added to each tube up to 10% (vol/vol) and incubated at 37°C for 4 h. 100 μl gas was taken out from each tube and analyzed using a gas chromatograph (Agilent Technologies 7890A). Nitrogen-fixing ability was expressed as μmol C_2_H_4_ produced h^-1^ g ^-1^ of fresh nodules. Each measurement was repeated at least three times.

## Results

### Identification of the TlpA1 Transmembrane Chemoreceptor in *A. caulinodans* ORS571

**FIGURE 1:**
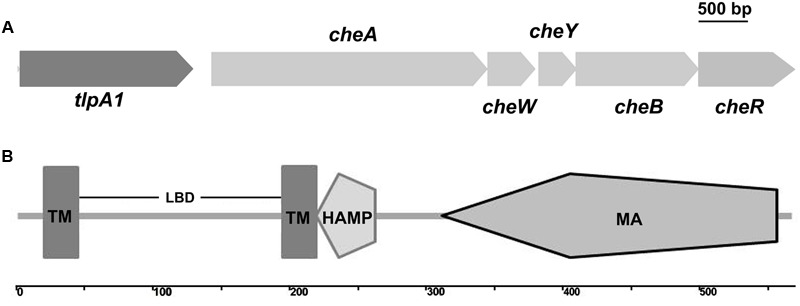
Schematic diagram showing the genome neighborhood of the *tlpA1* gene and domain architecture of the TlpA1. **(A)** Genome neighborhood of the *tlpA1* (AZC_0660) gene. Arrows indicate the direction of transcription. *cheA* (AZC_0661), *cheW* (AZC_0662), *cheY* (AZC_0663), *cheB* (AZC_0664), *cheR* (AZC_0665). **(B)** Domain architecture of the predicted TlpA1 protein, as defined by the SMART database: TM, transmembrane regions; LBD, periplasmic ligand binding domain; HAMP domain (histidine kinases, adenylyl cyclases, methyl binding proteins, and phosphatases); MA, methyl-accepting chemotaxis-like domain.

We used MiST2 and SMART databases and identified a chemotaxis gene cluster in the genome of *A. caulinodans* ORS571. This cluster encodes homologs of known chemotaxis proteins, which we termed as *che* gene cluster (Jiang et al., 2016b). This unique *che* gene cluster contains *cheA, cheW, cheY, cheB*, and *cheR* genes with a chemotaxis receptor, which we named *tlpA1* (transducer-like protein in *A. caulinodans*), located upstream of *cheA* and predicted to be transcriptionally co-oriented (**Figure 1A**). TlpA1 is predicted to be a ∼60 kDa protein. N-terminal domain of TlpA1 consists of two transmembrane helices (TM) and a periplasmic ligand-binding domain (LBD), while the C-terminal region consists of a HAMP and a highly conserved MA domain (**Figure 1B**).

Similarity searches using BLASTP revealed that TlpA1 is homologous to chemoreceptors from closely related alpha-proteobacteria such as *Azorhizobium doebereinerae, Afifella, Ancylobacter*, and *Chelatococcu* (Supplementary Figure S2A). Sequence similarity searches with the N-terminal periplasmic ligand-binding region of TlpA1 followed by multiple sequences alignment of related proteins showed that this region is found exclusively in the N-terminal variable extracellular regions of chemoreceptors from various distantly related bacterial species (Supplementary Figure S2B). The presence of a conserved LBD domain in various classes of chemoreceptors suggests a possible fitness advantage to bacteria in different environments. Interestingly, 30 other chemoreceptors of *A. caulinodans* ORS571 also contain two transmembrane regions in the N-terminal region, and HAMP and chemoreceptor signaling domains in their C-terminal regions as predicted by MiST2 and SMART. However, sequence similarity searches using BLASTP indicated that neither the entire TlpA1 protein sequence nor the N-terminal periplasmic region is homologous to the other 30 transmembrane chemoreceptors (Supplementary Figure S2).

### The Δ*tlpA1* Mutant Is Impaired for Chemotaxis

**FIGURE 2:**
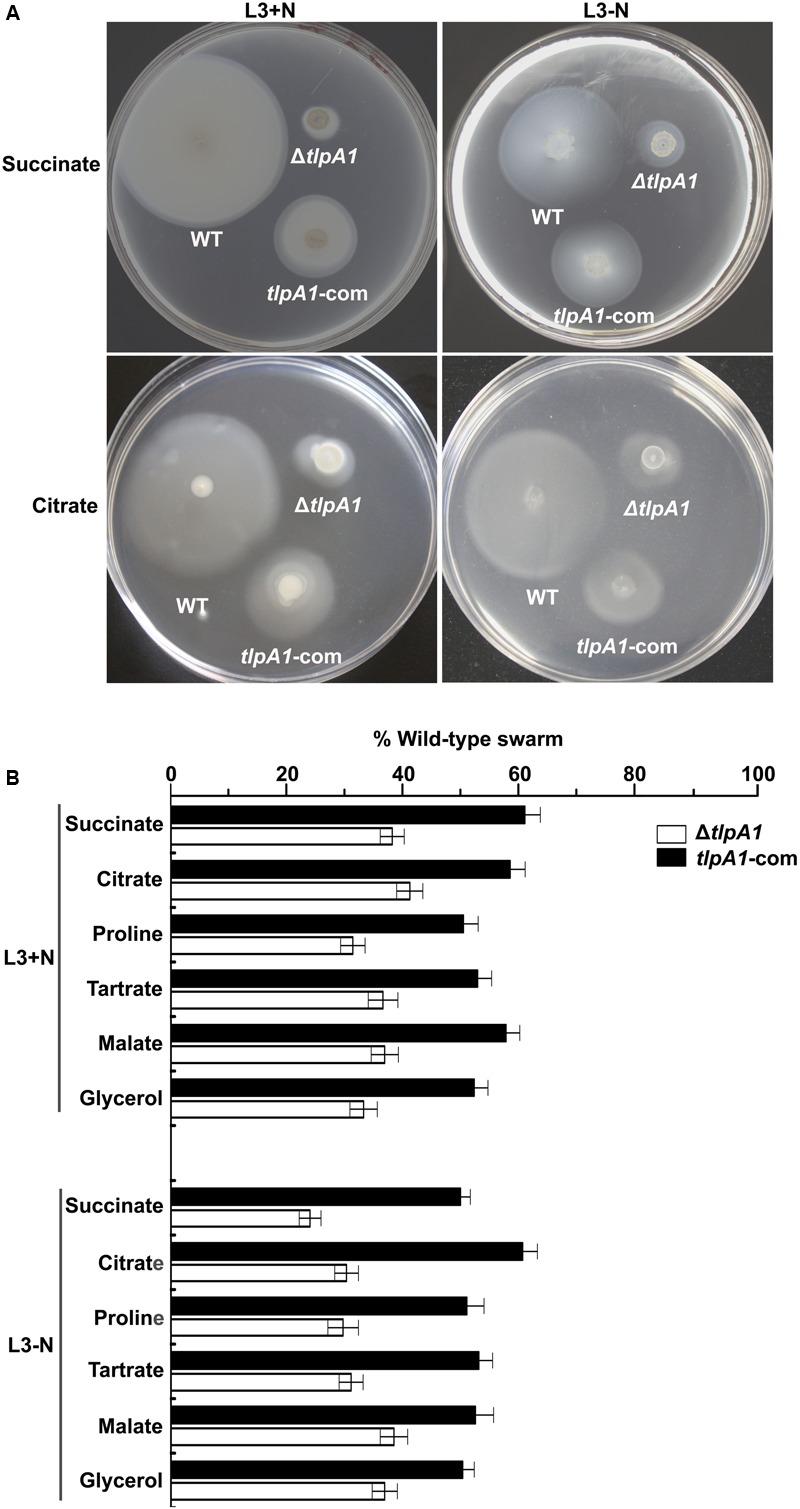
The Δ*tlpA1* is deficient for chemotaxis. **(A)** Chemotactic behaviors of the wild-type (WT) *A. caulinodans* ORS571 (WT), Δ*tlpA1* strain and complemented strains (*tlpA1*-com) were compared by soft agar plate assay. Pictures shown are representative plates for these strains on L3+N/-N with succinate and citrate as sole carbon sources. **(B)** Average diameters on L3+N/-N plates were expressed as percentages relative to that of the wild-type strain (defined as 100%). Error bars represent standard deviations (SD) from the mean calculated from at least three repetitions.

To determine the role of the chemotaxis receptor TlpA1 in *A. caulinodans* ORS571, we generated strain containing a *tlpA1* deletion mutant (named Δ*tlpA1*). Soft agar plates were used to assess chemotaxis towards carbon sources such as succinate, citrate, tartrate, malate, proline, and glycerol, which are strong attractants of *A. caulinodans* (Jiang et al., 2016a). The Δ*tlpA1* mutant was significantly impaired (∼25 to 40% of wild-type) in chemotaxis towards all carbon sources tested, regardless of presence or absence of combined nitrogen (**Figures 2A,B**). We complemented the Δ*tlpA1* strain with a plasmid carrying the wild-type *tlpA1* gene. The chemotaxis defect of the Δ*tlpA1* mutant was partially rescued by expression of the wild-type *tlpA1* gene driven by its native promoter (**Figures 2A,B**).

**FIGURE 3:**
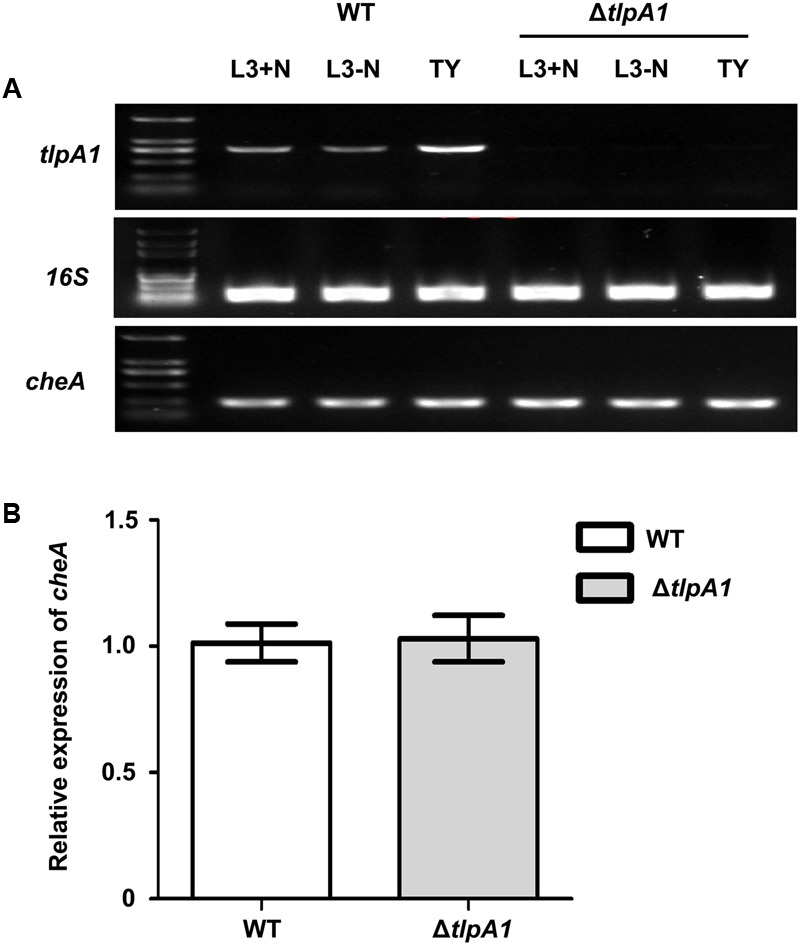
Expression of the *cheA* gene in the wild-type and Δ*tlpA1* strains. **(A)** The *cheA* and *tlpA1* genes were detected by PCR using the WT and Δ*tlpA1* cDNA as templates; *16S rRNA* gene was amplified as a positive control. **(B)** Expression of the *cheA* gene in the WT and Δ*tlpA1* strains was detected by qRT-PCR, with *16S rRNA* gene as internal standard. All data were derived from three independent experiments and are expressed as mean and SD. The WT and Δ*tlpA1* strains were grown in TY medium (OD_600_ = 0.8).

Since the *tlpA1* gene is located directly upstream of *cheA* and transcribed in same direction (**Figure 1A**), we determined whether deletion of *tlpA1* affected the *cheA* promoter region and thus *cheA* gene expression. We thus compared *cheA* gene expression in the wild-type and Δ*tlpA1* strains and found that *cheA* was expressed in both strains (**Figures 3A,B**). Furthermore, there was no difference in growth rates of the mutant and wild-type cells in TY medium (Supplementary Figure S3A) or in L3 minimal medium (Supplementary Figure S3B). These data suggested that the diminished diameters observed on the soft agar plate are related to defects in chemotaxis.

### Motility of the Δ*tlpA1* Mutant Is Not Impaired

**FIGURE 4:**
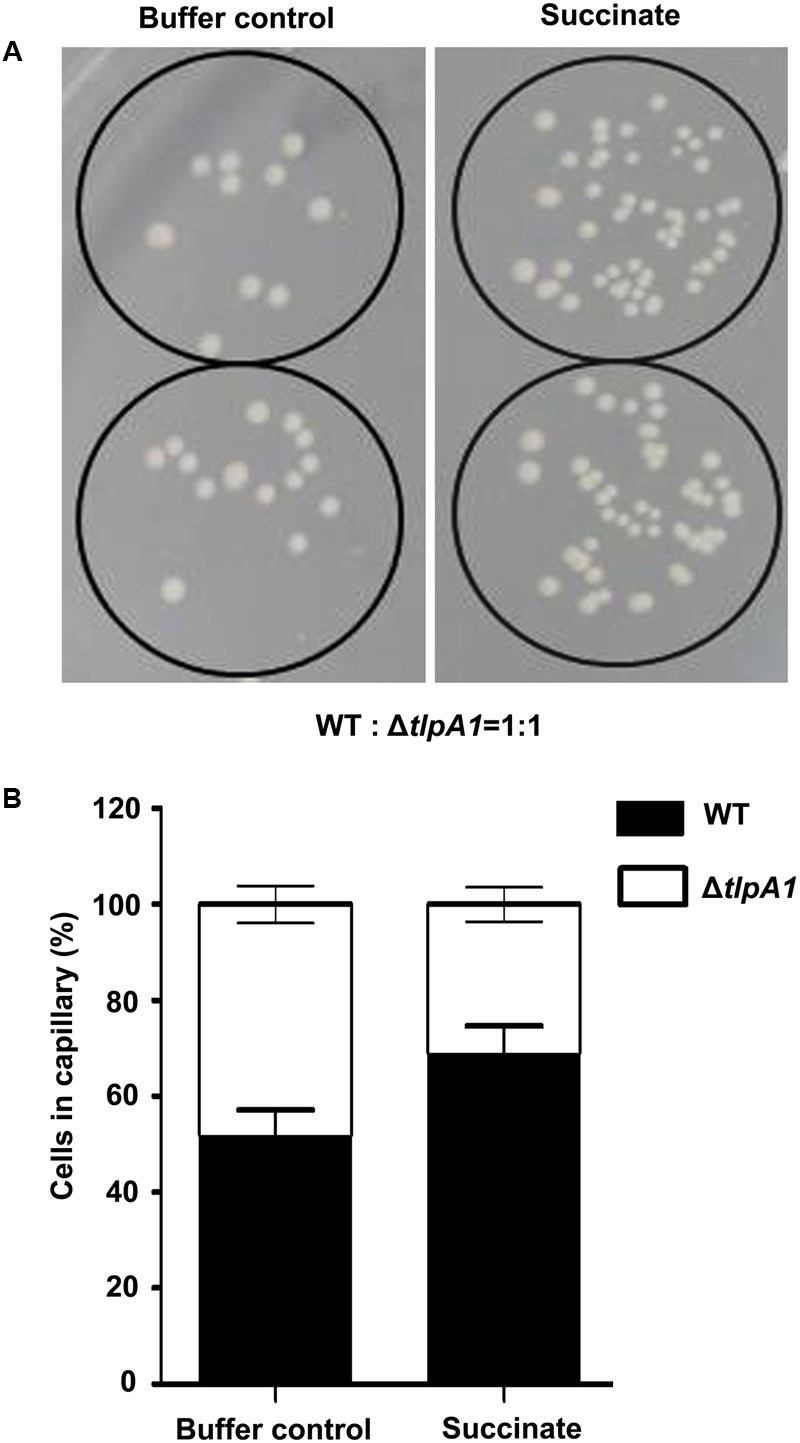
Competitive quantitative capillary chemotaxis assays of the WT and Δ*tlpA1* mutant. **(A)** TY agar plates used for cell counting in the quantitative capillary chemotaxis assay. The capillaries filled with buffer control (left) or 10 mM succinate as the carbon source (right) were inserted into wells containing the WT and Δ*tlpA1* (1:1 ratio) bacterial suspension mixture. **(B)** Statistical analysis of the WT and Δ*tlpA1* mutant cell ratios in the capillary. The error bars represent the SE of data from three independent experiments.

To further assess chemotaxis and motility of the Δ*tlpA1* mutant, competitive quantitative capillary assays of the wild-type and Δ*tlpA1* strains were performed. In this assay, capillaries filled with chemoeffector are immersed into a bacterial suspension, which causes cells to preferentially swim into the capillary filled with chemoattractant. Buffer without chemoattractant was used as a control (**Figure 4A**). After this assay, the number of colony forming units in the capillaries was determined. For competitive quantitative capillary chemotaxis assay, a bacterial suspension containing a mixture of the wild-type and Δ*tlpA1* strains at an approximate 1:1 ratio was used. **Figure 4A** shows competitive quantitative capillary chemotaxis assay with buffer and succinate as the attractants for the wild-type and Δ*tlpA1* mutant. The cell count of the Δ*tlpA1* from capillaries filled with buffer was similar to that of the wild-type (about 1:1) (**Figure 4B**). This observation indicates that swim motility behavior of the free-swimming cells is not affected by the Δ*tlpA1* mutant. However, cell count of the Δ*cheA* mutant from capillaries filled with buffer was significantly less than that of the wild-type cells (Supplementary Figure S4). With succinate as the attractant, the wild-type cell count was almost twice that of the Δ*tlpA1* mutant (**Figure 4B**). This data shows that the Δ*tlpA1* mutant is impaired in chemotaxis and that TlpA1 is essential for chemotaxis but not for cell motility in *A. caulinodans* ORS571.

### The Δ*tlpA1* Mutant Is Impaired for Biofilm Formation

**FIGURE 5:**
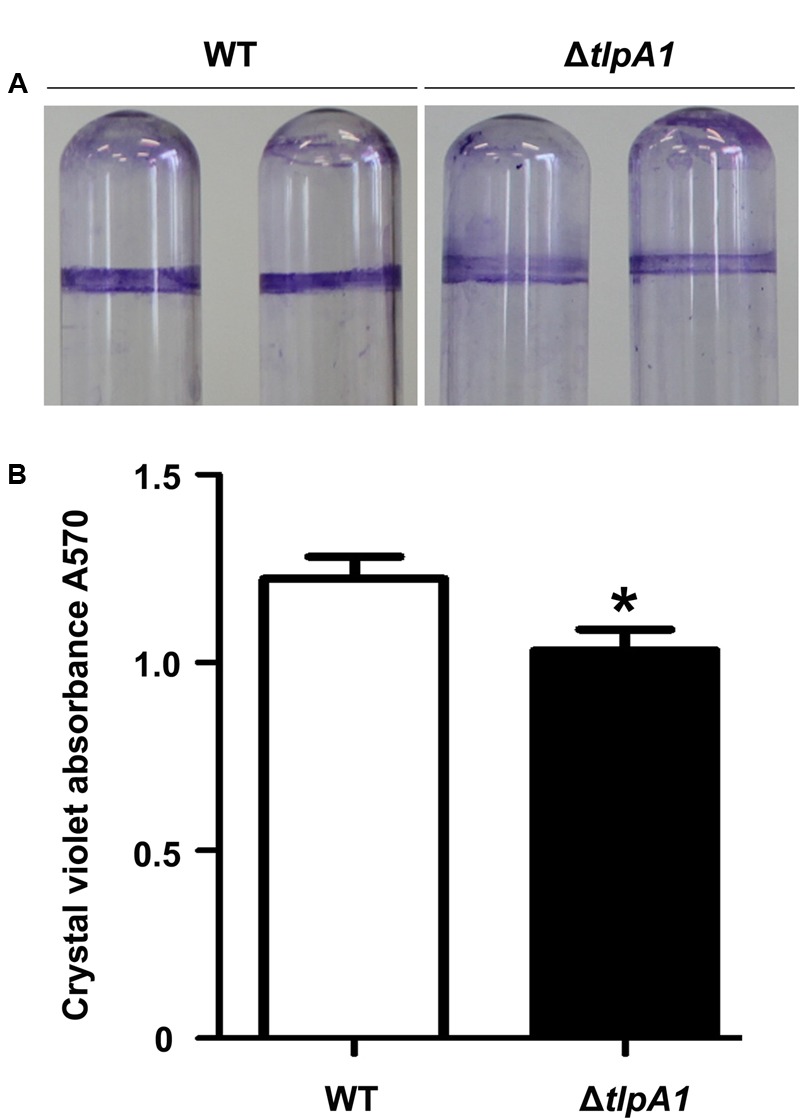
The Δ*tlpA1* mutant is impaired for biofilm formation. **(A)** Biofilm morphology upon crystal violet staining of the WT and Δ*tlpA1* mutant. **(B)** Biofilm formation was quantified by crystal violet staining method. The error bars represent SE of means from five independent experiments. Asterisks (^∗^) indicate statistically significant differences (*P* < 0.05) between the wild-type and Δ*tlpA1* mutant.

Chemosensory systems have been previously implicated in regulation of biofilm formation (Hickman et al., 2005). To test whether deletion of *tlpA1* affected biofilm formation, we compared biofilm formation ability of the Δ*tlpA1* with the wild-type. Quantitative data reveal that biofilm formation in the Δ*tlpA1* mutant is less than that in the wild-type (**Figures 5A,B**). These data indicate that TlpA1 may directly modulate biofilm formation under certain growth conditions.

### The Δ*tlpA1* Is Impaired for Competitive Colonization and Nodulation of Host Plants

**FIGURE 6:**
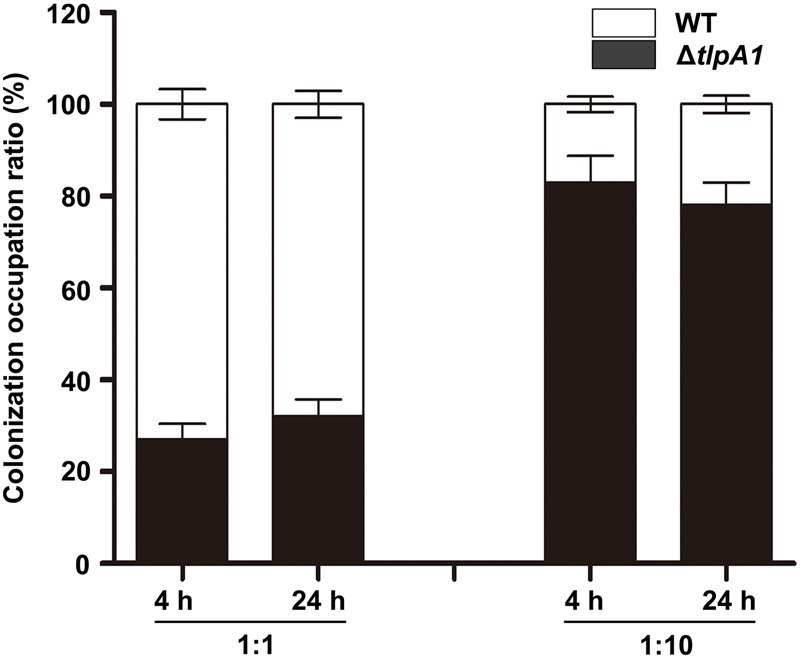
The Δ*tlpA1* is impaired for competitive colonization of *S. rostrata* root surfaces. Colonization ratios of the WT and Δ*tlpA1* strains were analyzed. The pattern of competitive colonization of the WT and Δ*tlpA1* strains for adsorption to the surface of roots of *S. rostrata* seedlings is shown at 4 h and 24 h after inoculation. Mixed inocula at 1:1 and 1:10 ratios were used and analyzed by PCR with the primer pair TlpA1F and TlpA1R. Similar results were obtained in three independent experiments. The error bars represent standard errors of mean calculated for three independent experiments.

To investigate the role of TlpA1 in symbiosis, the colonization ability on surface of roots was assessed by a quantitative assay. When inoculated individually, cell number of the Δ*tlpA1* strain re-isolated from *S. rostrata* root surfaces was not significantly different from that of the wild-type (Supplementary Figure S5). This result suggested that the Δ*tlpA1* is able to colonize on root surface. Surface-sterilized seedlings were co-inoculated with the wild-type ORS571 and Δ*tlpA1* at approximately 1:1 and 1:10 ratios and cell numbers of the wild-type and Δ*tlpA1* strains on the root surface of *S. rostrata* seedlings were determined after inoculation. The results indicated that colonization efficiency of the Δ*tlpA1* mutant was significantly reduced when compared to the wild-type strain (**Figure 6**). These data therefore suggest that the Δ*tlpA1* mutant affects competitive colonization of host root surfaces.

**FIGURE 7:**
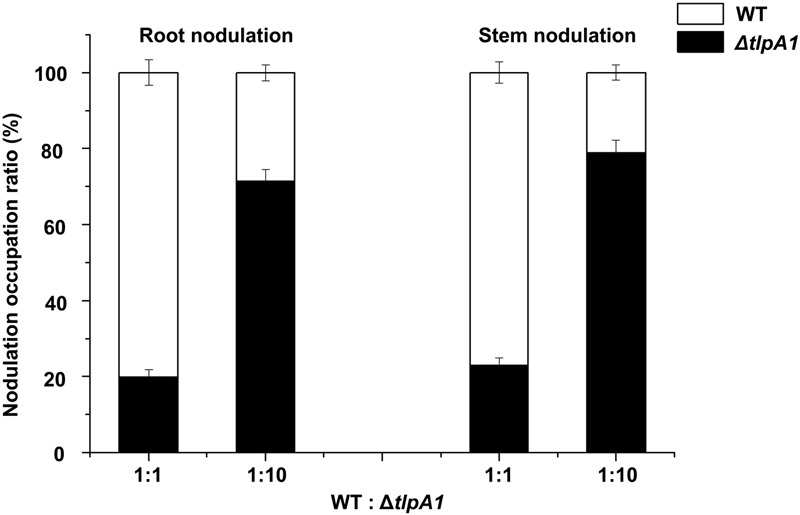
Competitive nodulation tests between the WT and Δ*tlpA1* mutant. Cell numbers of the Δ*tlpA1* mutant and WT were determined on root (left) and stem (right) nodules. The x-axis indicates ratios between the WT and Δ*tlpA1* mutant. Error bars represent standard errors of the mean calculated from three independent experiments.

Root colonization is an important step in the establishment of symbiosis between rhizobia and host plants (Benizri et al., 2001). To further test whether the Δ*tlpA1* mutant is defective for symbiosis and nodulation inducing ability in host, the wild-type and Δ*tlpA1* strains were inoculated individually or in combination onto roots and stems of *S*. *rostrata*. The nodule occupancy of each strain was then determined. As shown in Supplementary Figures S6A–C, the Δ*tlpA1* mutant induced functional nodules on roots and stems when inoculated alone, with no differences in nodule number and morphology as compared to the wild-type. Leghemoglobin of the stem nodules induced by the Δ*tlpA1* mutant was orange–brown 20 days post inoculation (Supplementary Figure S6B). This result indicates that TlpA1 does not affect leghemoglobin production of the host plant in mature nodules. Likewise, nitrogenase activities of the root and stem nodules formed by the Δ*tlpA1* strain were similar to the wild-type (Supplementary Figure S7). When we tested the ability of the mutant to induce nodule formation on roots in competition with the wild-type, we found that it was impaired in its ability to form nodules (**Figure 7**). The Δ*tlpA1* mutant could not successfully compete for nodule formation with the wild-type even at 10-fold excess. We also tested the ability of the mutant to form stem nodules in competition with the wild-type. The results were similar to those obtained for root nodules (**Figure 7**). Taken together, these results indicate that the TlpA1 chemoreceptor is implicated in the processes related to competitive nodulation.

## Discussion

Bacterial chemotaxis is initiated by transmembrane receptor proteins that undergo conformational changes upon ligand binding (Falke and Hazelbauer, 2001). Chemoreceptors link environmental stimuli to bacterial motility via regulation of a CheA/CheY two-component system. Our results demonstrate that deficiency of the transmembrane chemoreceptor TlpA1 results in obvious defects in chemotaxis towards multiple carbon sources, suggesting that it plays a major role in chemotaxis of *A. caulinodans* ORS571. The *tlpA1-*deficient strain has a phenotype similar to that of a *mcpB* mutant in *Rhizobium leguminosarum* that also affects chemotaxis towards all carbon sources tested (Yost et al., 1998). The *tlpA1* and *mcpB* mutant strains appear to have a generalized defect in chemotaxis, although the reasons remain unclear. Interestingly, this chemotaxis phenotype does not completely conform to the *E. coli* model where a given chemoreceptor is responsive to a specific set of ligands, indicating that the role of certain chemoreceptors in alpha-proteobacteria may substantially differ from those of *E. coli*. In addition, compared to the loss of a soluble cytoplasmic chemotaxis receptor IcpB in *A. caulinodans* ORS571 (Jiang et al., 2016a), loss of TlpA1 more detrimental to chemotaxis. Furthermore, the swim motility behavior of free-swimming cells is not affected in the Δ*tlpA1* mutant (**Figure 4**) and is different from that of the *cheA1*-deficient strain (Supplementary Figure S4).

In the *tlpA1*-deficient strain, cells are impaired for chemotaxis in various carbon source chemical gradients, including creation of gradients on the swim plates or on the host root. Root adsorption and nodulation tests suggested that the *tlpA1*-deficient strain could colonize roots and induce nodules when inoculated alone. Furthermore, the Δ*tlpA1* mutant can induce functional nodules on roots and stems without any significant difference in nodule number, morphology and nitrogenase activities as compared to the wild-type. However, the competitive colonization and nodulation efficiency of Δ*tlpA1* was significantly lower than that of the wild-type (**Figure 7**). These findings point to an important role of chemotaxis in host root colonization and nodulation. Similarly, an *Azospirillum brasilense* mutant lacking an energy-sensing chemoreceptor is severely compromised for wheat root surface colonization (Greer-Phillips et al., 2004). In addition, nodulation competition experiments with *R. leguminosarum* suggested that two chemoreceptors MCPB and MCPC are important for pea nodulation (Yost et al., 1998). It has been speculated that chemotaxis may play a role in competition between rhizobia strains in legume rhizosphere. Some studies have shown that non-motile or non-chemotactic mutant strains cannot compete effectively with the wild-type strain (Ames and Bergman, 1981; Siuti et al., 2011). We found that cell motility of the Δ*tlpA1* mutant is similar to that of the wild-type. Therefore, colonization and competitive nodulation of host plants appear to be affected by chemotaxis.

Root colonization ability is mainly determined by chemotaxis and biofilm formation (Hölscher et al., 2015). Although there is evidence suggesting that chemosensory pathways are involved in biofilm formation process, it remains unclear as to how chemoreceptors and signals modulate this process. In this study, we showed that biofilm formation ability of the Δ*tlpA1* mutant was reduced when compared to that of the wild-type. In *Pseudomonas putida* KT2440, four chemoreceptor mutants had significantly altered biofilm phenotypes. Similarly, a strain carrying the mutation PP1488 showed a slight reduction in bacterial spread radii on soft agar as well as had a reduced capacity to form biofilms (Corral-Lugo et al., 2016). Further work will be done to understand how biofilm formation affected by chemoreceptors.

In summary, our data demonstrate that the Δ*tlpA1* mutant is impaired for chemotaxis, biofilm formation, and competitive colonization and nodulation when tested on roots and stems. Our results suggest that impaired competitiveness to host roots apparently results in reduced nodule occupancy. These findings indicate that environmental signals or other stimuli trigger chemotaxis by the transmembrane receptor TlpA1 of *A. caulinodans* cells towards roots and stems of *S. rostrata*.

## Author Contributions

WL and ZX conceived and designed the experiments. WL, JY, YS, XL, performed the experiments. YL and ZZ provided technical support. WL and ZX were responsible for the drafting the manuscript. All authors contributed to manuscript revisions.

## Conflict of Interest Statement

The authors declare that the research was conducted in the absence of any commercial or financial relationships that could be construed as a potential conflict of interest.
