# Supplementary material for: Azorhizobium caulinodans Transmembrane Chemoreceptor TlpA1 Involved in Host Colonization and Nodulation on Roots and Stems
Source: Front Microbiol. 2017 Jul 13;8:1327. doi: 10.3389/fmicb.2017.01327 (PMC5508009; doi:10.3389/fmicb.2017.01327)
Supplement: Supplementary file 1 [file Data_Sheet_1.DOCX]

***Supplemental* *Material:***

***Azorhizobium caulinodans* Transmembrane Chemoreceptor TlpA1 Involved in Host Colonization and Nodulation on Roots and Stems**

Wei Liu^1^, Jinbao Yang^2^, Yu Sun^1,3^, Xiaolin Liu^1,3^, Yan Li^1^, Zhenpeng Zhang^1,3^, Zhihong Xie*^1^

*Correspondence: Zhihong Xie, Yantai Institute of Coastal Zone Research, Chinese Academy of Sciences, 17 Chunhui Road, Laishan District, Yantai, Shandong, China

e-mail: zhxie@yic.ac.cn

**Figure S1. Physical maps of Δ*tlpA1* and Δ*cheA*.** Physical maps of flanking regions used for construction of the Δ*tlpA1* (A) and Δ*cheA* (B) mutants. Arrows indicate the direction of transcription.**
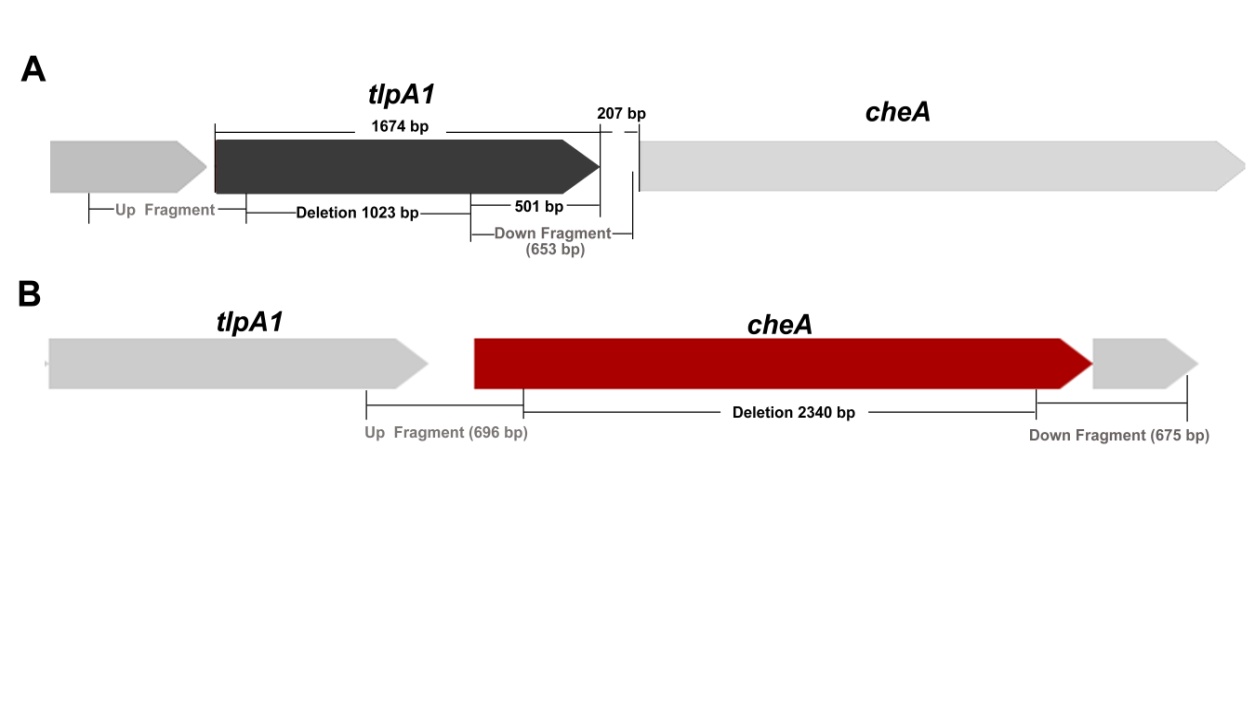
**

**Figure S2. Multiple sequence alignment analyses of TlpA1 and the N-terminal periplasmic region from *A. caulinodans* ORS571 and related sequences.** (A) Multiple alignment of TlpA1 and related homologous protein sequences. (B) Multiple alignment of the N-terminal periplasmic ligand binding domain of TlpA1 and related homologous amino acid sequences. Multiple alignments were constructed using ClustalW of BioEdit program. Identical residues are highlighted in black, and the consensus for multiple alignments (85% threshold) is highlighted in gray. Each sequence in the alignment is identified by its GenBank identification number and by the abbreviated name of organism. Abbreviations: MCP, methyl-accepting chemotaxis protein; Azor, *Azorhizobium doebereinerae*; Afif, *Afifella*; Ancy, *Ancylobacter*; Chel, *Chelatococcus*; Ensi, *Ensifer*; Sino, *Sinorhizobium*; Rhiz, *Rhizobium*; Devo, *Devosia*.**
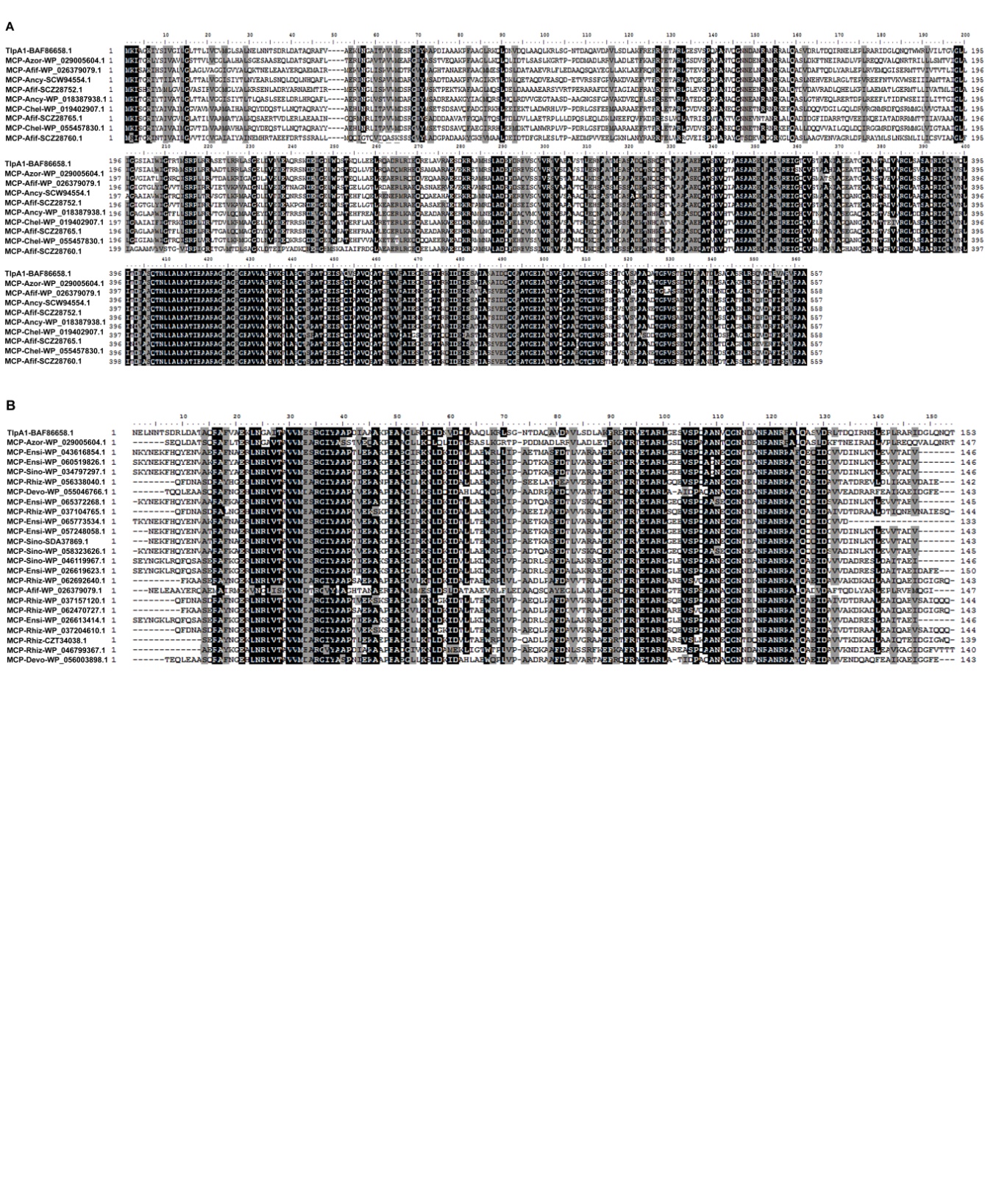
**

**Figure S3. Growth rates of the wild-type and Δ*tlpA1* strains are not different.** (A) Growth curves of the wild-type (WT) and Δ*tlpA1* in TY liquid medium. The Optical density (OD_600_) was measured at different time points. (B) Growth curves of the WT and Δ*tlpA1* strain in L3 liquid medium. Cells were grown in L3 minimal medium with 10 mM succinate as sole carbon source and 10 mM NH_4_Cl as nitrogen source.**
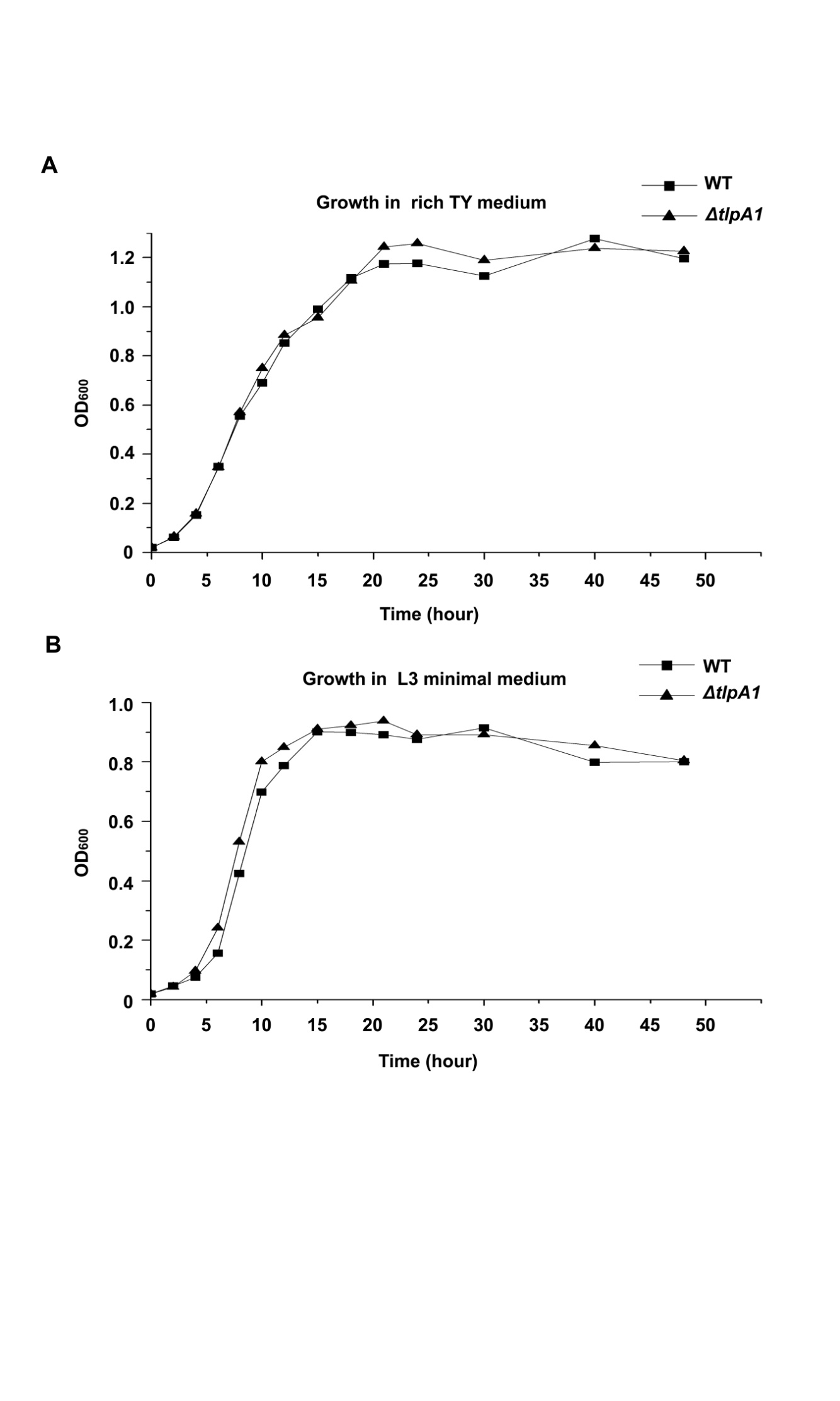
**

**Figure S4. Competitive quantitative capillary chemotaxis assays with the wild-type and Δ*cheA* mutant.** Statistical analysis of the wild-type (WT) and Δ*cheA* mutant cells ratios in control capillary filled with buffer. Error bars represent standard errors (SE) from data of three independent experiments.


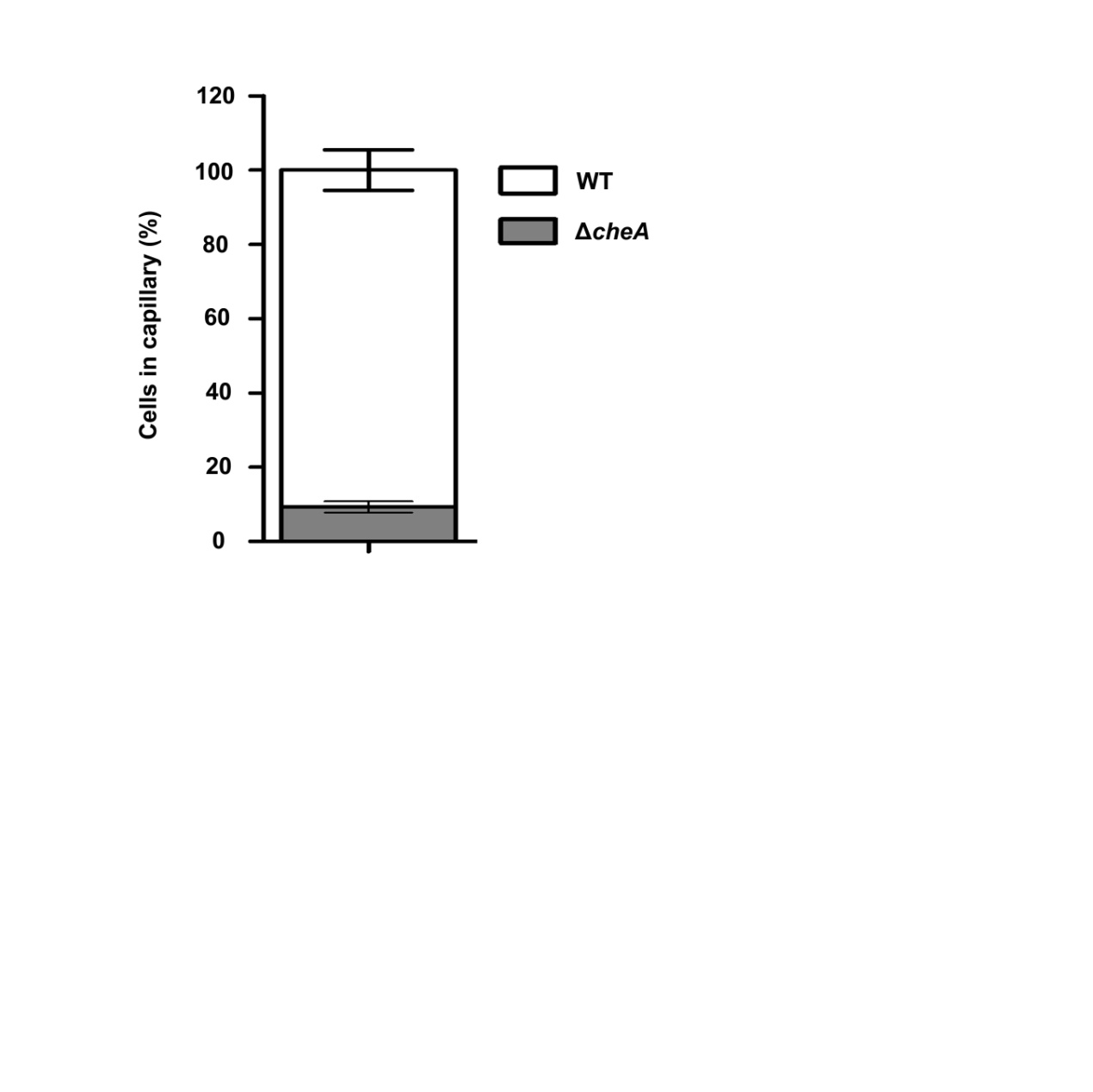


**Figure S5. Root surface colonization of the wild-type and Δ*tlpA1* strains.** Bacteria were re-isolated from the root surface of *S.* *rostrata* seedlings after inoculation for 4 h and 24 h. TY agar plates were used for cell counting.

**
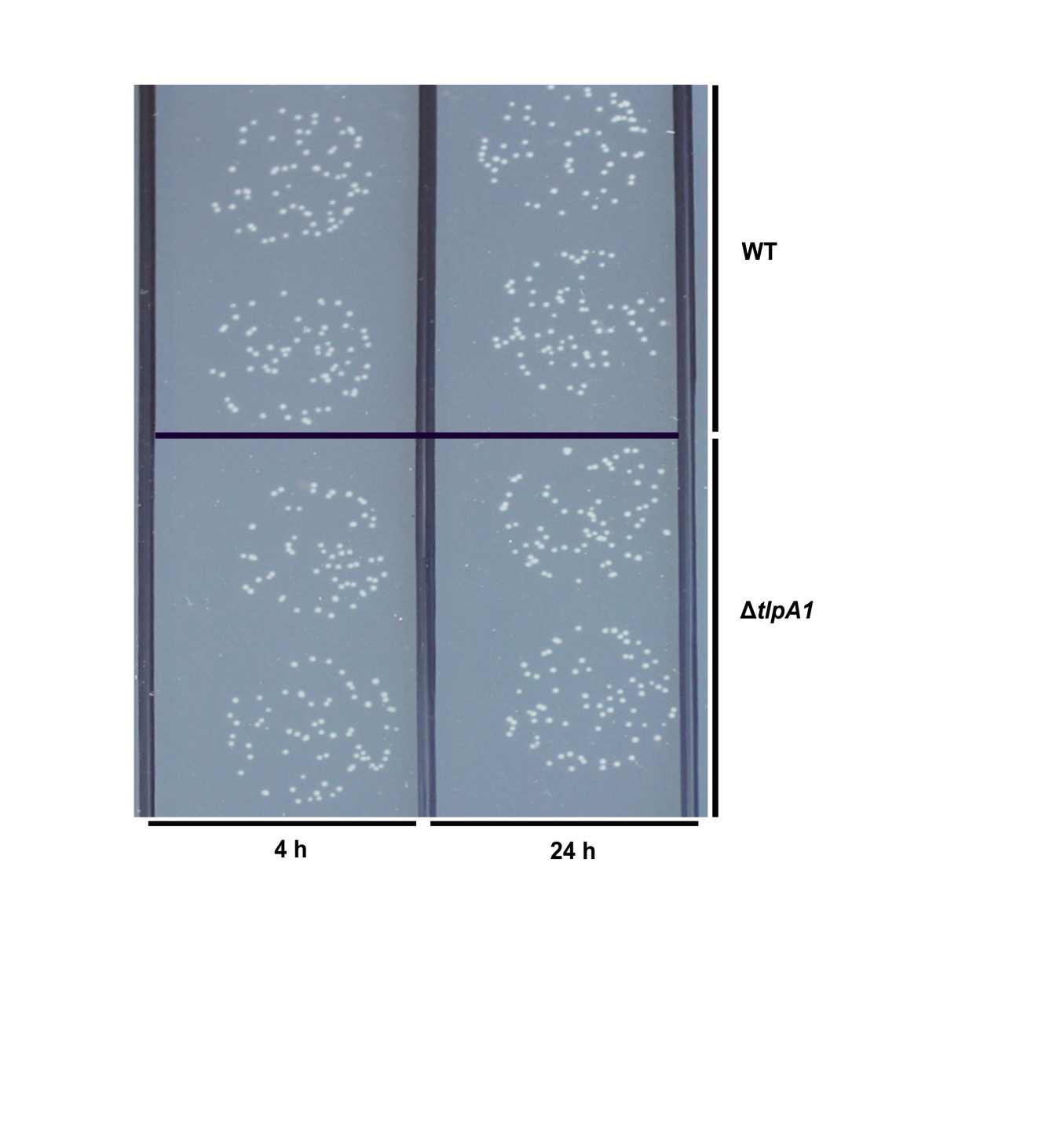
**

**Figure S6. Nodulation tests with the wild-type and Δ*tlpA1* strains.** (A) Typical appearances of stem and root nodules induced by the wild-type (WT) and Δ*tlpA1* mutant. (B) Leghemoglobin from stem nodules shows a characteristic orange-brown color. (C) Root nodules induced by the WT (left) and Δ*tlpA1* (right).

**
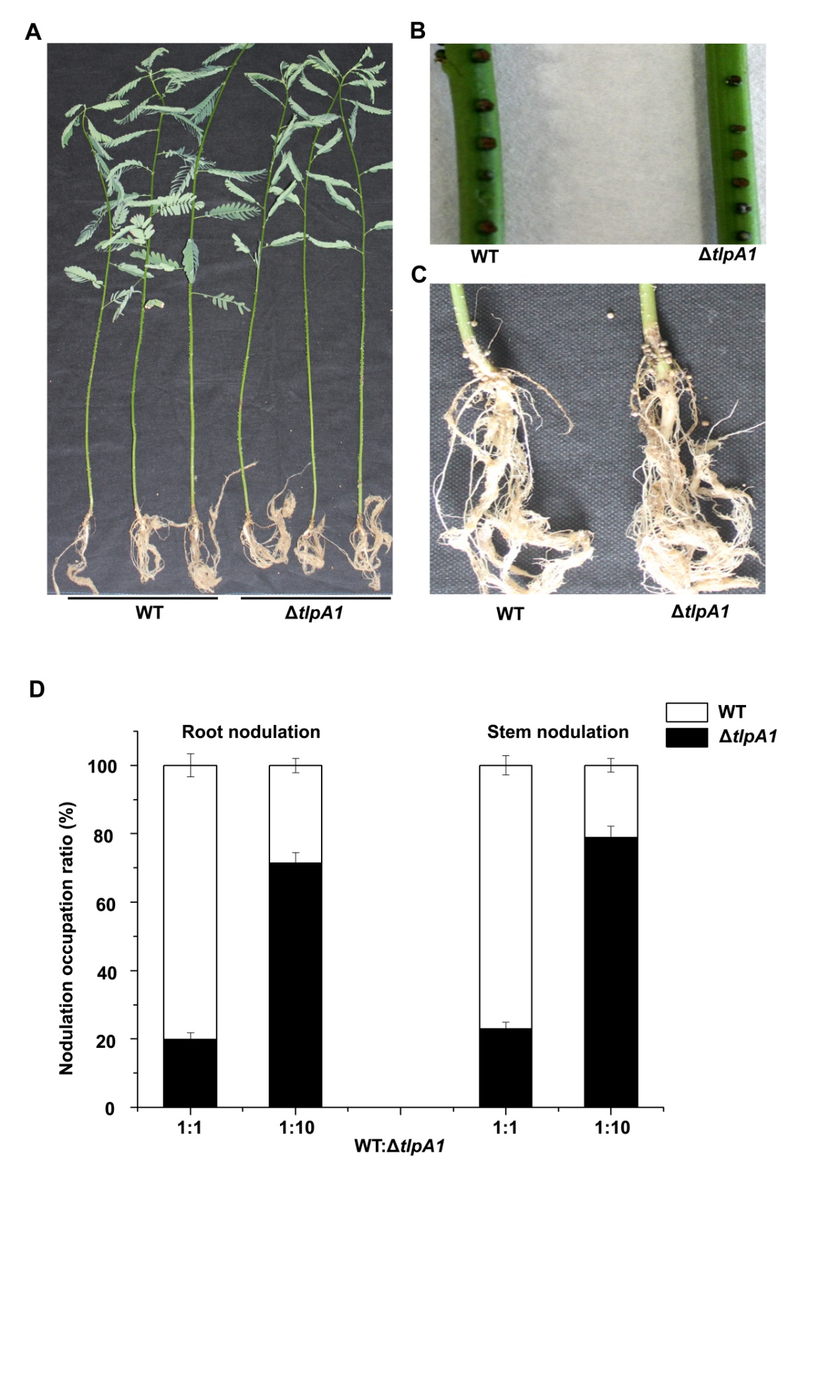
**

**Figure S7. Acetylene reduction activities of root and stem nodules induced by the wild-type and Δ*tlpA1* strains***.* The error bars represent standard errors (SE) of mean calculated from six independent experiments.

**
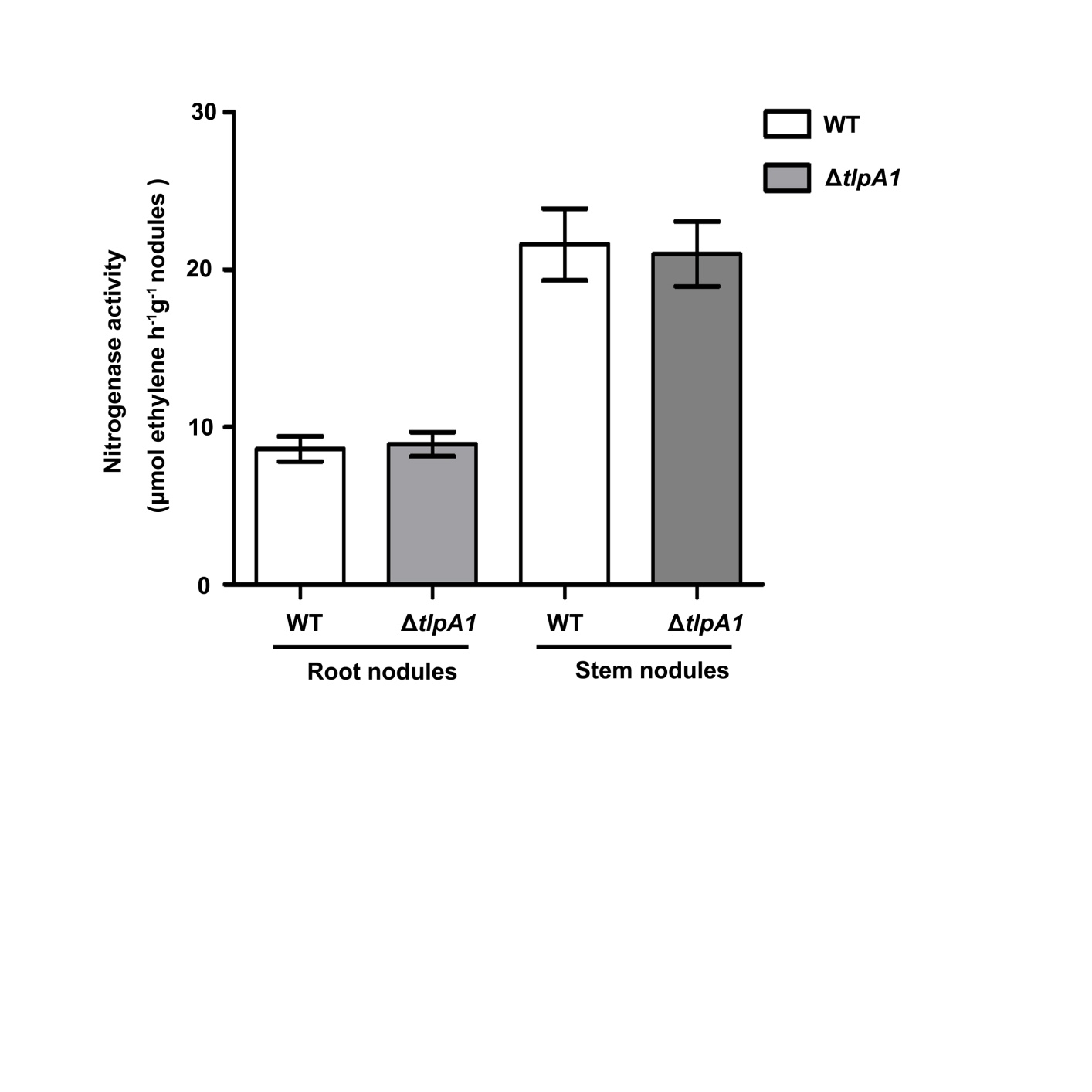
**

**Table S1. Strains and plasmids used in this study.**

| **Strain or plasmid** | **Relevant properties** | **Source or reference** |
| --- | --- | --- |
| **Strain :**  *A. caulinodans* ORS571 | Wild-type strain, Amp^r^, Nal^r^ | (Dreyfus et al., 1988) |
| Δ*tlpA1* | ORS571 derivative, *tlpA1* deletion mutantion, Amp^r^, Nal^r^, Gen^r^ | This study |
| *tlpA1*-com | Δ*tlpA1* strain harbouring the pBBR1MCS-2-*tlpA1* plasmid, Amp^r^, Nal^r^, Gen^r^, Kan^r^ | This study |
| Δ*cheA* | ORS571 derivative, *cheA* deletion mutant, Amp^r^, Nal^r^, Gen^r^ | This study |
| *E. coli* DH5a | *F^-^* SupE44Δ*lac*U169 (*φ80 lacZ*Δ*M15*) hsdR17 *rec*A1 *endA1 gyrA96 thi-1 relA1* | Transgen |
| **Plasmids :**  pCM351 | Mobilizable allelic exchange vector, Amp^r^, Gen^r^ | (Marx and Lidstrom, 2002) |
| pBBR1MCS-2 | Broad-host-range cloning vector, Kan^r^ | (Kovach et al., 1995) |
| pRK2013 | Helper plasmid, ColE1 replicon, Tra+, Kan^r^ | (Figurski and Helinski, 1979) |
| pCM157 | IncP plasmid that expresses Cre recombinase, Tet^r^ | (Marx and Lidstrom, 2002) |
| pBBR-*tlpA1* | pBBR1MCS-2 with *tlpA1* ORF and 737-bp upstream promoter region, Kan^r^ | This study |

Amp^r^, ampicillin resistance; Nal^r^, Nalidixic acid; Gen^r^, gentamicin resistance; Kan^r^, kanamycin resistance, Tet^r^ tetracycline resistance.

**Table S2. Primers used in this study.**

| **Primer** | **Sequence (5’-3’)*** | **Purpose** |
| --- | --- | --- |
| TlpA1UF-KpnⅠ | GGGGTACCGTTCGTGGTCATCGGCGTC | Δ*tlpA1* mutant construct |
| TlpA1UR-NdeⅠ | GGAATTCCATATGCTCAGCCACGAAGGCGCG | Δ*tlpA1* mutant construct |
| TlpA1DF-ApaⅠ | TGGGCCCGTGGTGCAGCTCATCACTGAC | Δ*tlpA1* mutant construct |
| TlpA1DR-SacⅠ | CGAGCTC CAGGCCCTTCGGACATTCC | Δ*tlpA1* mutant construct |
| TlpA1comF-HindⅢ | CCCAAGCTTCAGCCTGCTGGAGCTCAGC | *tlpA1*-com construct |
| TlpA1comR-BamHⅠ | CGGGATCCGAAGGTGGTCCGTCCTCAG | *tlpA1*-com construct |
| CheAUF-NsiⅠ | CCAATGCATGGACACCATCCGCAAGATTGAC | Δ*cheA* mutant construct |
| CheAUR-NdeⅠ | AATTCCATATGGATCTTGTTCGAACTTGAC | Δ*cheA* mutant construct |
| CheADF-AgeⅠ | GACCGGTGTGGTCAGCGATCTGGAGATG | Δ*cheA* mutant construct |
| CheADR-SacⅠ | GC TCTAGAGTCGAACTTGGCGATGTAGTC | Δ*cheA* mutant construct |
| TlpA1F | GCTCGATAACGTGGACCAAC | Validation of *tlpA1* |
| TlpA1R | CCGTCTGAACGTTGCTTGTA | Validation of *tlpA1* |
| TlpA1QF | TATCTCGGACACCATCCGCA | qRT-PCR for *tlpA1* |
| TlpA1QR | GACGAAAGTGTCGACCTGGCT | qRT-PCR for *tlpA1* |
| 16SQF | GAGAGTTTGATCCTGGCTCAG | qRT-PCR for *16S rRNA* |
| 16SQR | CAGCTACTGATCGTCGCCTTGG | qRT-PCR for *16S rRNA* |
| CheAF | CAGGAAGACTCCGAATACAAGGT | Validation of *cheA* |
| CheAR | GATCATCTCGATGTTCGAGCG | Validation of *cheA* |
| CheAQF | TGGATGATCTGCTGCGCGAGT | qRT-PCR for *cheA* |
| CheAQR | CCATCAGCGTCTCGGCCGCATG | qRT-PCR for *cheA* |

*****Engineered restriction sites are underlined.
